# Antimicrobial effects of microwave plasma-activated water with skin protective effect for novel disinfectants in pandemic era

**DOI:** 10.1038/s41598-022-10009-1

**Published:** 2022-04-08

**Authors:** Hye Ran Lee, Yun Sang Lee, Young Suk You, Jin Young Huh, Kangil Kim, Yong Cheol Hong, Chul-Ho Kim

**Affiliations:** 1 Department of Otolaryngology, School of Medicine, Ajou University, 164 World-Cup Street, Yeongtong-gu, Suwon, 16499 Republic of Korea; 2 Department of Otolaryngology-Head and Neck Surgery, Catholic Kwandong University International St. Mary’s Hospital, Incheon, 22711 Republic of Korea; 3 Plarit Co., Ltd., 443 Samnye-ro Samnye-eup, Wanju-gun, Jeollabuk-do 565-701 Republic of Korea; 4 ICD Co., Ltd., 274 Manse-ro, Daedeok-myeon, Anseong-si, Gyeonggi-do 17542 Republic of Korea; 5 Institute of Plasma Technology, Korea Institute of Fusion Energy, 814-2 Ohsikdo-dong, Gunsan, 573-540 Republic of Korea; 6 Division of Applied Technology Research, National Fusion Research Institute, 113 Gwahangno, Yuseong-gu, Daejeon, 305-333 Republic of Korea

**Keywords:** Biochemistry, Microbiology, Health care

## Abstract

Skin antiseptics have important implications for public health and medicine. Although conventional antiseptics have considerable antimicrobial activity, skin toxicity and the development of resistance are common problems. Plasma-treated water has sterilization and tissue-regenerative effects. Therefore, the aim of this study was to identify whether plasma-activated water (PAW) manufactured by our microwave plasma system can be used as a novel antiseptic solution for skin protection. PAW was produced by dissolving reactive nitrogen oxide gas using microwave plasma in deionized water. The antibacterial effects of PAW against *Staphylococcus aureus, Escherichia coli, Pseudomonas aeruginosa, Bacillus cereus,* and *Salmonella typhimurium* and effective concentrations were investigated by a solid agar plate assay. The factors mediating the effects of PAW were evaluated by the addition of reactive species scavengers. Cytotoxicity and cell viability assays were performed to examine the protective effect of PAW on normal skin cells. PAW exhibited excellent sterilization and no toxicity in normal skin cells. Experiments also confirmed the potential of PAW as a sanitizer for SARS-CoV-2. Our findings support the use of PAW as an effective skin disinfectant with good safety in the current situation of a global pandemic.

## Introduction

Skin antiseptics are chemical agents applied to the skin to destroy pathogenic microorganisms and reduce the risk of infectious diseases, including surgical site infections^1^. They are used as handwashing products for general public health and are essential agents for medical procedures, such as pre-surgical and procedural skin preparation^2^. In particular, with the outbreak of new infectious diseases such as coronavirus disease 2019 (COVID-19), the importance of handwashing is being emphasized more than ever in any place. For daily use by individuals, including children, antiseptics must show good efficacy and safety.

Commercially available skin antiseptics contain alcohol, povidone-iodine, chlorhexidine, hydrogen peroxide (H_2_O_2_), and silver compounds as main components. They each have their own strengths and weaknesses; however, they all show rapid sterilizing effects via various cascades affecting multiple non-specific cellular components in a broad spectrum of pathogens^3,4^. However, this cytotoxicity not only affects pathogens but also affects normal skin cells. In normal skin tissues, it results in a loss of regenerative potential and hydrophilicity by the denaturation of constituent proteins and intercellular lipids in skin cells^5,6^. These adverse effects on normal cells cause chronic side effects, such as irritant contact dermatitis and acute allergic reactions to active ingredients. Users exhibit various signs and symptoms, thereby affecting compliance with skin hygiene practices^3,7^. Damaged skin weakens the cleaning effect of antiseptics and increases the likelihood of the transmission of pathogenic microorganisms, leading to the spread of nosocomial infections and public health issues^2^. In addition to these issues, the unregulated routine use of typical antiseptics triggers the generation of multi-drug resistant pathogens and epidemic outbreaks^4,8^. Against this background, it is essential to develop novel antimicrobial agents with a different mechanism of action from those of existing antiseptics.

In the past few decades, several anti-infective bioactive strategies, including plasma, have been developed. Plasma is regarded as the fourth state of matter produced by ionization by applying sufficient thermal energy to the gaseous state. It has vigorous reactivity, while releasing excited molecules including free radicals and electrons, and reactive oxygen and nitrogen species (RONS), and other components such as ultraviolet (UV) radiation and photons^9,10^. Plasma is an innovative technology with applications in various fields, such as material processing, agriculture, environmental sciences, and medicine^10^. In particular, in the biomedical field, by its direct application to living tissues or indirect application through the development of new biomaterials, plasma can be used to regulate the viability and activity of various pathogens and target cells^11^.

As the applications of plasma have expanded, plasma-activated water (PAW) created by the addition of plasma to liquid media has been investigated^12^. PAW exhibits a similar efficacy to that of gas plasmas, while preserving the structural integrity of targets during treatment and is therefore advantageous for in vivo applications^13^. Liu et al*.* reported that active reactive species show better mouse skin penetration in liquid-type plasma than in gas-type plasma^14^. The sterilizing effects of plasma species generated in bulk water or plasma-liquid interfaces have been actively studied in the medical field^15,16^. The broad antimicrobial spectrum of PAW has been reported, against bacteria, viruses, spores, biofilms, and even fungi^10,17^. The biochemical activity of PAW is based on changes in physicochemical properties, such as the pH and redox potential, due to the vigorous action of RONS, produced by the reaction of highly activated plasma gas and water^18,19^. Since potentially harmful chemicals are not required for the production and activity of these effectors, PAW could be an innovative sterilizing solution without hazardous effects on the environment^19,20^.

In the present study, we constructed a new microwave plasma system and characterized the physicochemical properties and bactericidal effects of PAW generated using this system. Furthermore, we investigated whether the major effectors of our microwave PAW maintained bactericidal activity for several months and did not exert harmful effects on normal skin cells. Finally, severe acute respiratory syndrome coronavirus 2 (SARS-CoV-2) antiviral effect was investigated to determine the utility of PAW in the current pandemic situation caused by COVID-19. Taken together, the objective of this study was to develop a novel skin sanitizing solution that exhibits antimicrobial effects against COVID-19 for a sufficient period of time and overcomes the shortcomings of typical antiseptics by protecting normal skin.

## Results

### Generation of reactive species and PAW

**Figure 1:**
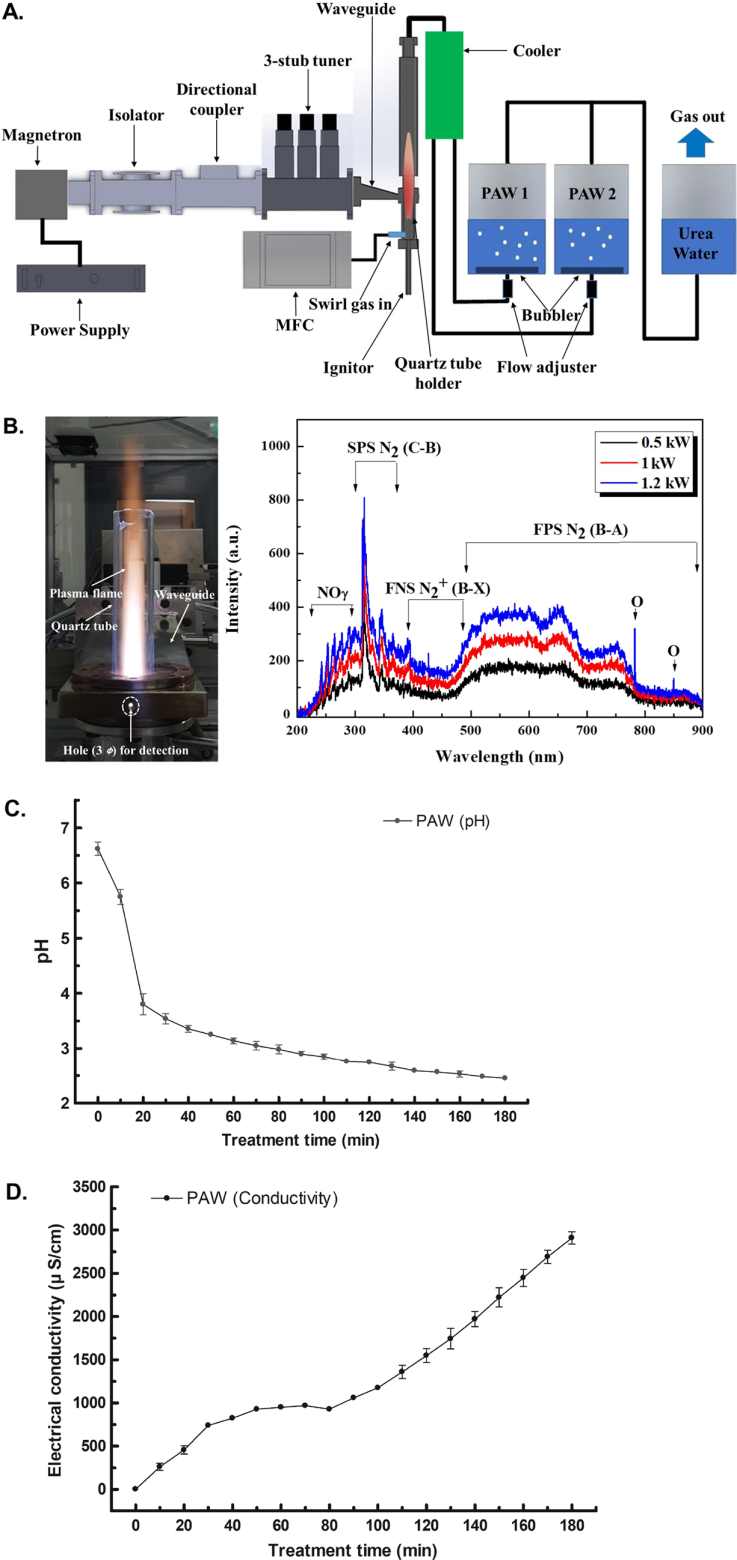
System for the generation of atmospheric microwave plasma-activated water (PAW) and real-time measurements of the pH and conductivity of PAW. (**A**) Schematics of the 2.45 GHz microwave plasma system and plasma-activated water system. (**B**) Optical emission spectra showing the chemical species produced from the plasma system operated at different powers. (**C**) The pH of PAW monitored for 3 h during microwave plasma treatment. (**D**) Plasma-activated water (PAW) conductivity increased gradually as the plasma treatment time increased. All data points represent means ± standard deviations of three replicate measurements.

PAW was produced by a microwave plasma system, generating nitrogen and oxygen species from air as the primary reactive species. The design of the device and the detailed technique are described in the Materials and Methods section (Fig. 1A). High-temperature air plasma, such as microwave discharge, can produce reactive oxygen species (ROS) and reactive nitrogen species (RNS)^19,21^. In general, the torch plasma flame becomes longer as the electrical power increases^22^. The plasma flame in Fig. 1B heats the quartz tube, which in turn emits its own light, revealing plasma emission spectra at different powers. As mentioned earlier, the air plasma flame can produce chemically active species, particularly excited nitrogen and oxygen atoms. To identify various excited plasma species generated by the air plasma torch, optical emission spectroscopy (Ocean Optics HR4000CG-UV-NIR, 300 grooves/mm) was employed over a wide wavelength range of 200–900 nm. The emission spectra of the air plasma torch, as shown in Fig. 1B, were mainly dominated by the presence of excited nitrogen molecules and oxygen atoms, showing different intensities at microwave powers of 0.5, 1, and 1.2 kW. The recorded spectra consisted of various molecular and atomic nitrogen and/or oxygen species [NO, N_2_ (SPS, second positive system), N_2_^+^ (FNS, first negative system), and N_2_ (FPS, first positive system) at 200–300 nm, 300–400 nm, 400–500 nm, and 500–900 nm, respectively, as well as O atoms at 777 nm and 844 nm]^23^. As shown in Fig. 1B, increasing the microwave power induced an important increase in emission from the NOγ system at 200–300 nm, suggesting an improvement in the dissociation mechanisms of molecular nitrogen and oxygen. Nitric oxide derivatives produced by the microwave plasma torch system might lead to the formation of the RONS liquid phase by reaction with O and OH derivatives in water^24^.

### Physicochemical properties of PAW

**Figure 2:**
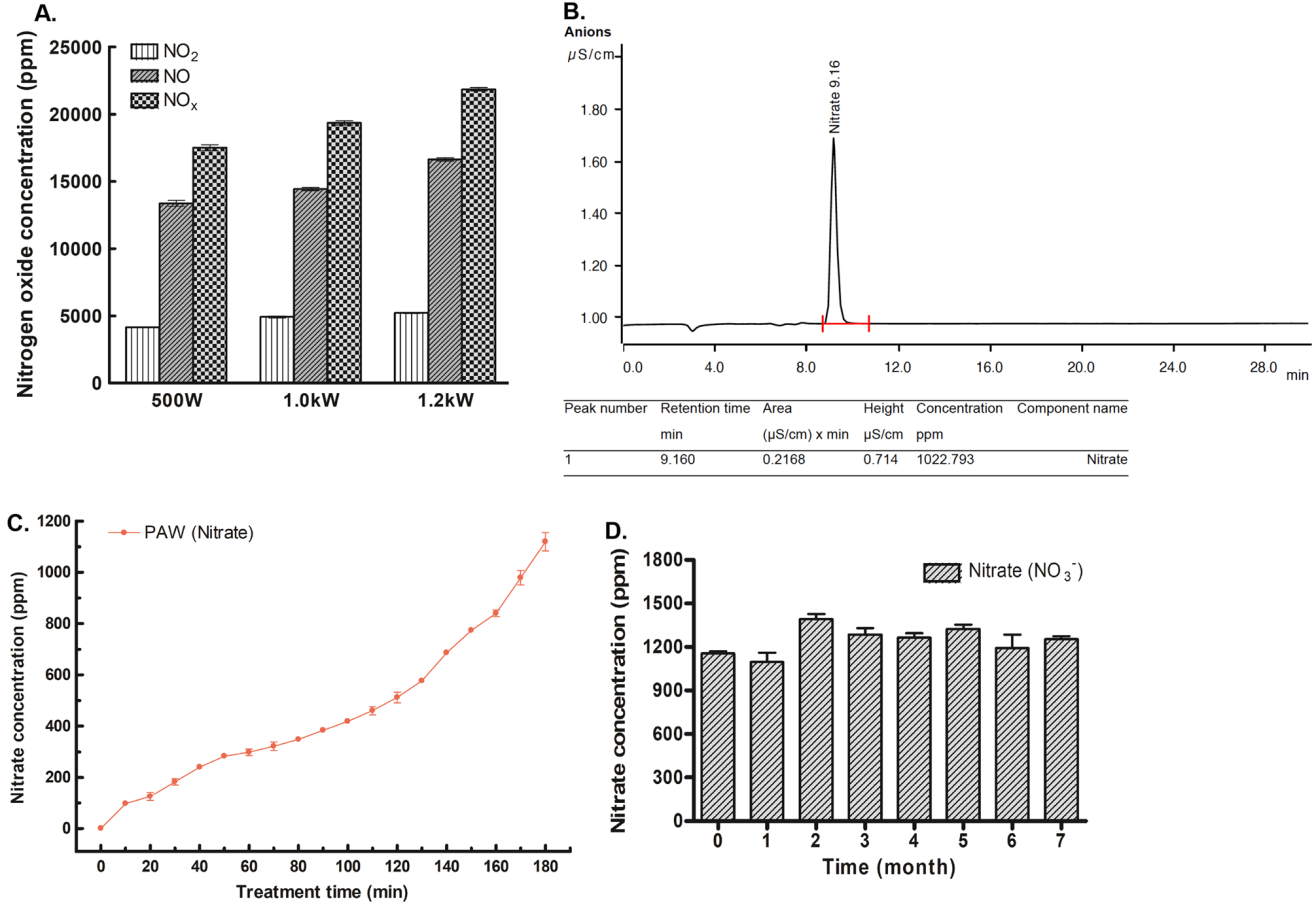
Measurement of nitrogen oxide produced by microwave plasma and nitrate concentrations in plasma-activated water (PAW). (**A**) The concentrations of total nitrogen oxide (NOx), nitric oxide (NO) and nitric dioxide (NO_2_) were assessed at microwave powers of 500 W, 1.0 kW, and 1.2 kW. As the power increased, species generation increased. (**B**) Only nitrate anion (NO_3_^−^) was detected by ion chromatography in PAW. (**C**) Changes in the nitrate (NO_3_^−^) concentration of PAW over 3 h. (**D**) The nitrate (NO_3_^−^) concentration in PAW remained steady at 1095–1390.5 ppm for 7 months.

The RONS reaction with deionized water altered the pH and oxidation–reduction potential (ORP), thereby increasing the conductivity of PAW^18^. As shown in Fig. 1C,D, the PAW had a pH of 2.45 ± 0.08 and a rise in conductivity to 2909.00 ± 122.65 µs/cm at 3 h after the reactive species were dissolved in the solvent. To generate microwave plasma, 10 lpm of air at 500 W, 15 lpm of air at 1.0 kW, and 20 lpm of air at 1.2 kW were each injected to generate microwave plasma. Nitrogen oxide produced during plasma generation was measured, most of which was produced in the form of nitric oxide (NO) and nitric dioxide (NO_2_). As the microwave power increased, the amount of nitrogen oxide produced increased (Fig. 2A). Of the generated nitrogen oxides, NO is readily oxidized to NO_2_ in the presence of oxygen from air^25^, and NO_2_ can be hydrolyzed in liquid and dissolved in a solvent to create abundant RNS, such as nitrite (NO_2_^−^), nitrate (NO_3_^−^), and peroxynitrite (ONOO^−^)^19^. PAW was evaluated by ion chromatography, and only the nitrate form was detected among RNS generated at a retention time of 9.160 min (Fig. 2B). As the microwave plasma treatment time increased, the nitrate concentration in the PAW increased steadily, reaching 1119.3 ± 62.07 at 3 h (Fig. 2C). After the storage of PAW at room temperature, the nitrate concentration was maintained at 1000 ppm or more for 7 months (Fig. 2D).

### Anti-bacterial activity of PAW

**Figure 3:**
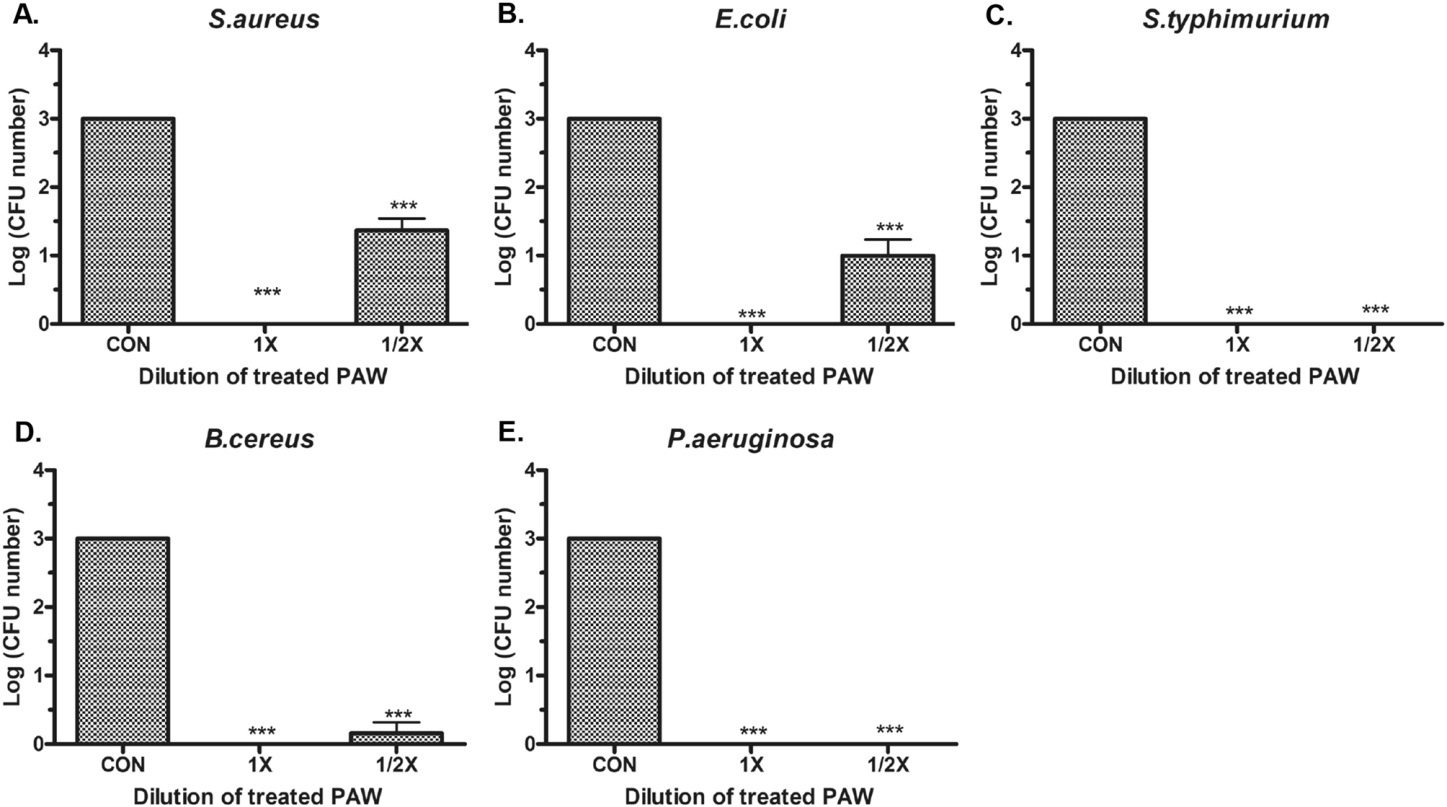
Bactericidal effect and effective concentration of plasma-activated water (PAW) for sterilization. (A) CFUs of (**A**) *S. aureus*, (**B**) *E. coli*, (**C**) *S.* Typhimurium, (**D**) *B. cereus,* and (**E**) *P. aeruginosa* treated with un-diluted (1 ×) and half-diluted (1/2 ×) PAW compared to those in untreated water samples (control, CON). Bacteria were treated for 5 min. Each value represents the average ± standard deviation of three replicate measurements. Three replicate measurements were used for statistical analyses. ***P < 0.001.

To evaluate the bactericidal effects of PAW, gram-positive *S. aureus* and *B. cereus* as well as pathogenic *E. coli*, *S.* Typhimurium, and *P. aeruginosa* able to colonize the skin were evaluated. In each cultured bacterial solution, the sterilizing effect of undiluted PAW and half-diluted solutions was analyzed 5 min after treatment. There was a 3 log CFU reduction in all five bacterial strains after treatment with undiluted PAW, equivalent to a 99.9% reduction in pathogens. Half-diluted PAW resulted in 1–2.5 log CFU reductions for *S. aureus* (Fig. 3A) and *E. coli* (Fig. 3B), an approximately 2.8 log reduction for *B. cereus* (Fig. 3D), and a nearly 3 log reductions for the other two bacterial strains, *S.* Typhimurium (Fig. 3C) and *P. aeruginosa* (Fig. 3E), indicating a bactericidal effect of at least 90% (Supplementary Fig. S1). Subsequent assays were performed using *S. aureus* and *E. coli*, which showed a reduction of less than 2.5 log CFU in half-diluted PAW, to determine the effective sterilization concentration of NO_3_^−^ (estimated as the minimum bactericidal concentration, MBC). Supplementary Fig. S2 shows that the MBC of NO_3_^−^ in PAW on *S. aureus* and *E. coli* was 662 ± 27.2 ppm, corresponding to a 99.99% bactericidal effect. As the NO_3_^−^ concentration in the undiluted PAW was maintained for more than 6 months, the sterilization effect for each bacterial strain was maintained. The sterilization effect was also maintained without colonies under various temperatures, such as − 80 °C, 4 °C, and room temperature as well as under direct sunlight (Supplementary Fig. S3).

### NO_3_^−^ isomer functioned as a major sterile effector of PAW

**Figure 4:**
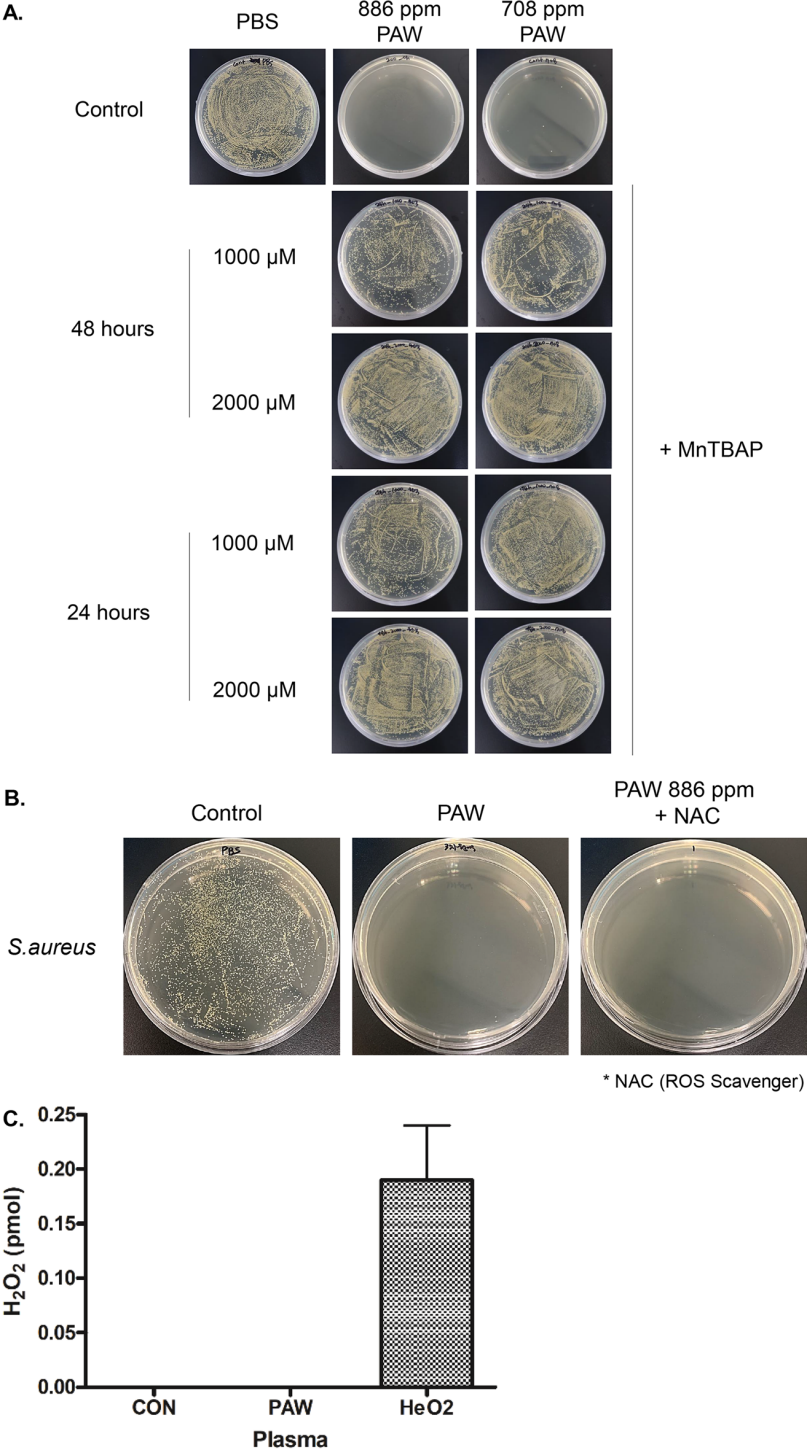
Peroxynitrate (ONOO^−^) and reactive oxygen species (ROS) scavengers experiment demonstrated bactericidal activity of ONOO^−^ in plasma-activated water (PAW). (**A**) Mn(III)tetrakis (4-benzoic acid) porphyrin (MnTBAP) as an ONOO^−^ scavenger added to PAW solutions with 886 and 708 ppm nitrate had little antibacterial activity, regardless of MnTBAP treatment time (24 and 48 h) and concentration (1000 and 2000 μM). (**B**) Bactericidal effect of *N*-acetyl-l-cysteine (NAC) as a ROS scavenger-supplemented PAW solution with 886 ppm nitrate against *Staphylococcus aureus* was the same as that of PAW. (**C**) Quantitative comparison of effects of H_2_O_2_ as ROS in PAW and HeO_2_ gas-type plasma with DW as a control. There was no detectable H_2_O_2_ in PAW or DW.

During the hydrolysis of primary nitric oxide species during the PAW manufacturing process generated ONOO^−^, an isomer of NO_3_^−^, and it has been consistently identified as important participant to microbial inactivation while having a strong oxidative potential^15,18^. To investigate whether ONOO^−^ can contribute to the bactericidal effect of PAW in this study, superoxide dismutase mimetic Mn(III)tetrakis (4-benzoic acid) porphyrin (MnTBAP), which acts as an ONOO^−^ scavenger and wide range oxidative redox signaling inhibitor^26,27^, was added to the PAW-treated culture medium of *S. aureus* and compared with the effect of PAW only. In the *S. aureus* culture medium treated with PAW, all bacterial colonies were eliminated, whereas the culture medium treated with MnTBAP and PAW did not show a bactericidal effect, regardless of the treatment time and the concentration of NO_3_^−^ (Fig. 4A). Along with RNS, including the NO_3_^−^ isomer, ROS are often presented as key components that play a dominant role in the biological activity of plasma^9,28^. To determine the effect of ROS on the sterilization efficacy of PAW, *S. aureus* survival was evaluated in groups treated with PAW and with *N*-acetyl-l-cysteine (NAC), a common ROS scavenger^29^, combined with PAW. There were no significant differences in the sterilizing effect between the groups treated with PAW only and with both PAW with NAC (Fig. 4B). The amount of H_2_O_2_, a major ROS produced by plasma^19^, was compared in PAW, gas-type HeO_2_ plasma, and DW, and there was no measurable H_2_O_2_ in the PAW and control groups (Fig. 4C). Therefore, NO_3_^−^ in isomer form with enhanced reactivity—but not ROS—significantly contributed to bactericidal effect of microwave PAW in this study.

### PAW did not have cytotoxicity in normal skin cells

**Figure 5:**
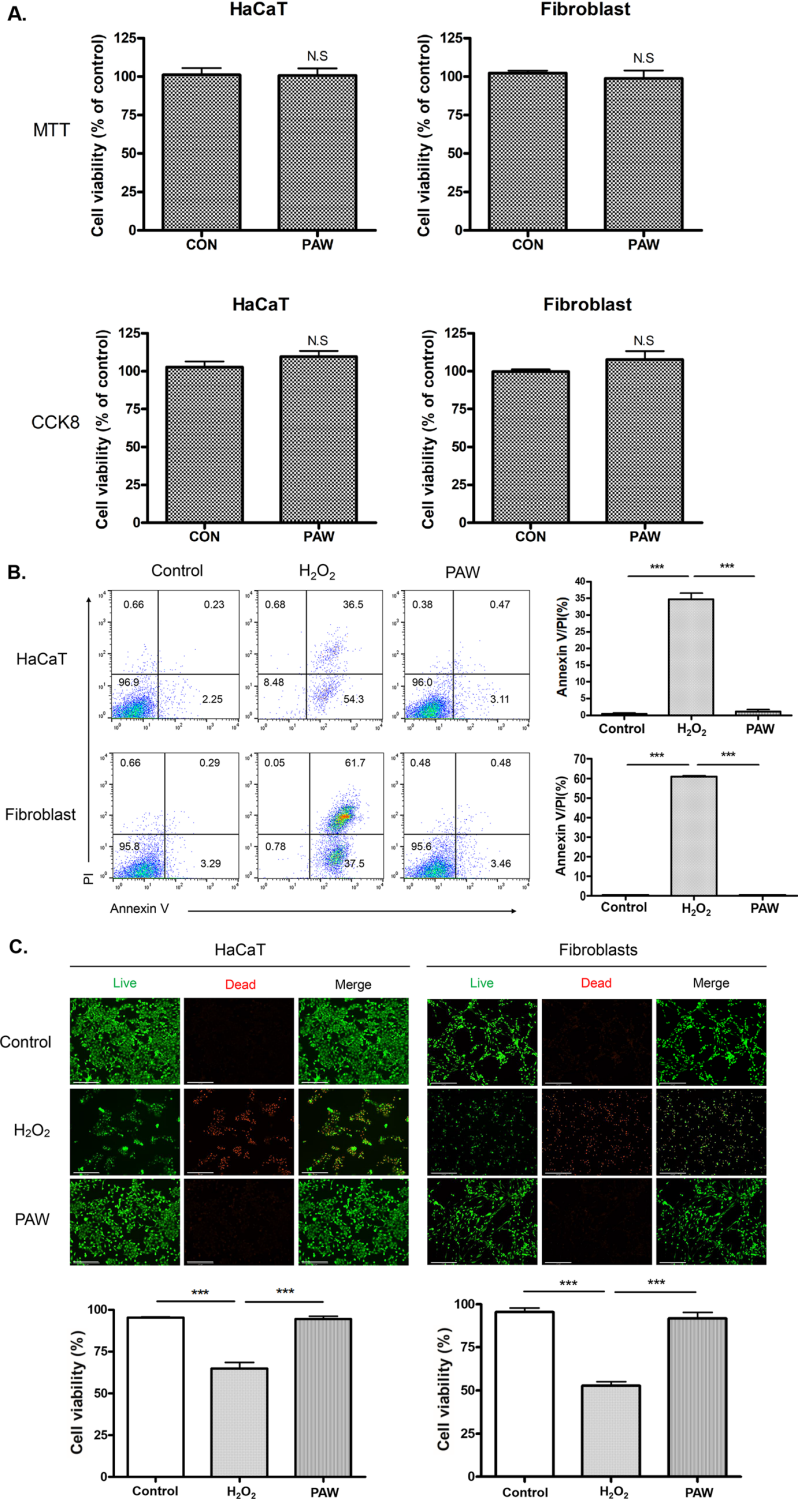
Skin protective effects of plasma-activated water (PAW) in vitro. (**A**) In normal skin cells (i.e., HaCaT and fibroblasts), cell viability was investigated by MTT and CCK8 assays after treatment with PAW and de-ionized water as a control (CON). There was no significant difference in cell viability between in the PAW-treated group and the control. (**B**) Annexin V/PI assay and quantification of HaCaT cells (upper) and Fibroblasts (lower) after treatment with DW as a control, PAW, and H_2_O_2_. (**C**) Live/dead cell assay evaluated by microscopy with a magnification of 200 × (scale bar, 200 μm). Live cells are marked with green-fluorescent calcein. No dead cells were visible in control and PAW-treated groups. H_2_O_2_-treated groups showed significant dead cells with red fluorescence in both HaCaT cells and fibroblasts. Three replicate measurements were used for statistical analyses. ***P < 0.001.

MTT, XTT, and CCK8 assays were used to evaluate the effects of PAW on normal skin cells (HaCaT and fibroblasts). There were no visual changes in the color of the culture medium between the PAW-treated group and the control group in MTT assay, and the consistent result was obtained in the CCK8 assay, which is more sensitive than MTT. Quantification by spectrophotometer system revealed no significant difference in either cell line between the PAW-treated group and the control group. The results of these assays showed that treatment with PAW in HaCaT cells and fibroblast did not induce cell death. In addition, PAW did not induce cell proliferation either. These results suggested that treatment of PAW did not affect in normal skin cells (Fig. 5A and Supplementary Fig. S4). Annexin V/PI toxicity of PAW was evaluated using HaCaT cells and fibroblasts. There were many spots in the upper right-hand corner, indicating dead cells in the H_2_O_2_-treated positive control group, whereas in the control and PAW-treated groups, most spots indicated normal cells in the lower left-hand corner (Fig. 5B). We performed a live/dead staining assessment to compare cell viability between the control and PAW groups. As shown in Fig. 5C, the viability of fibroblasts and HaCaT cells after PAW treatment was not significantly different from that of the control, and no dead cells (indicated by red fluorescence) were observed. Meanwhile, H_2_O_2_-treated cells as a positive control were significantly reduced and many dead cells were detected. According to the results of these cytotoxicity assays, PAW had no toxicity against normal skin cells.

### PAW had an antiviral effect on SARS-CoV-2

**Figure 6:**
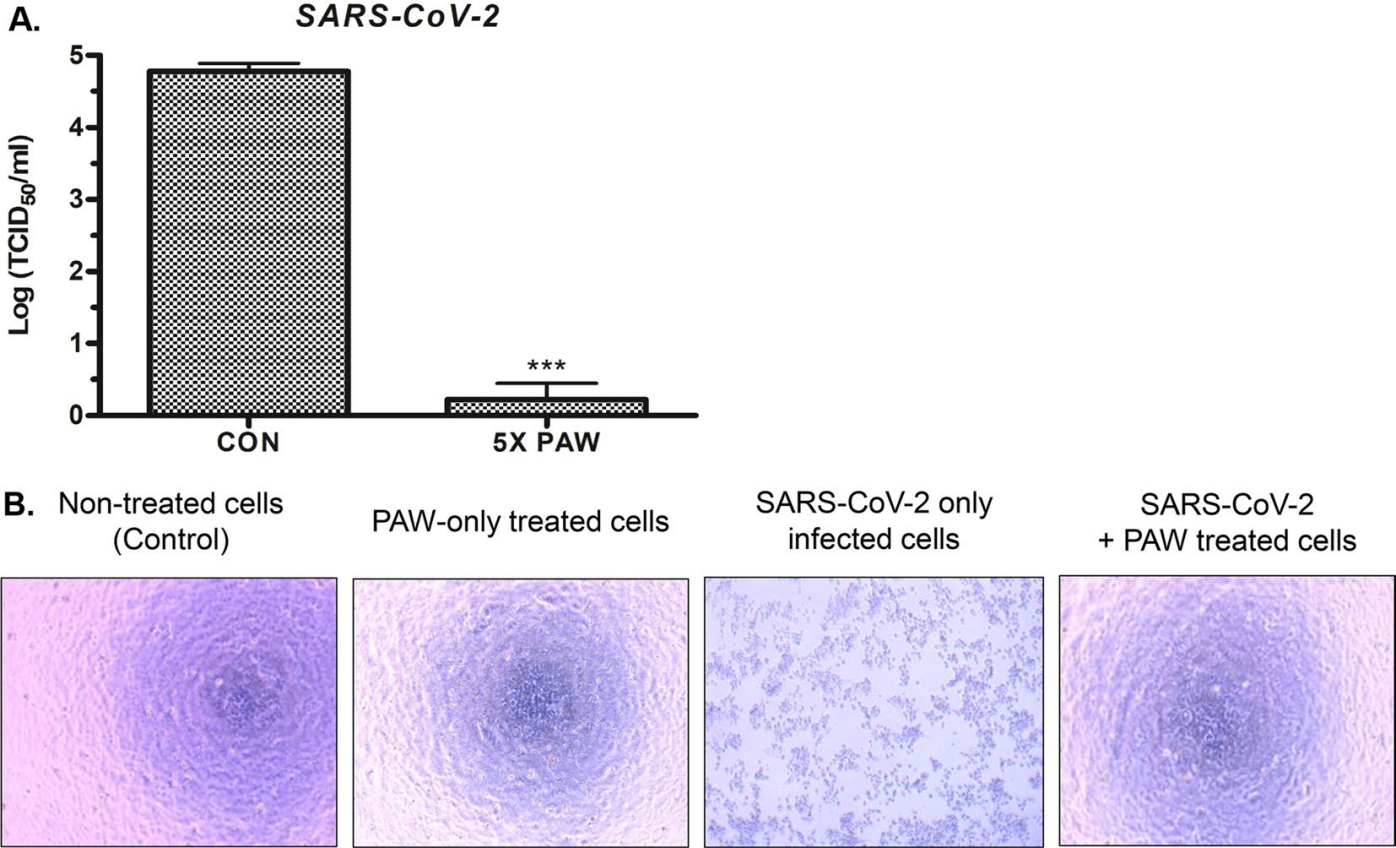
Effectiveness of plasma-activated water (PAW) treatment on SARS-CoV-2 (**A**) SARS-CoV-2 titer treated with concentrated (5 ×) PAW compared to those in PBS-treated samples (control, CON). Each sample was treated for 10 min. (**B**) Bright-field image of SARS-CoV-2 infected Vero-E6 cells showing a viral cytopathic effect (CPE). In the image of SARS-CoV-2 only infected cells, a significant decrease in the number of cells by cytopathic effect of SARS-CoV-2 was observed, compared with the other three images of normal Vero-E6 cells of non-treated, PAW-only treated, and treated with a mixture of SARS-CoV-2 and PAW groups. Each value represents the average ± standard deviation of three replicate measurements. Three replicate measurements were used for statistical analyses. ***P < 0.001.

Vero-E6 cells infected with SARS-CoV-2 and treated with a mixture of SARS-CoV-2 and 5 × PAW, in which a 5 times higher nitrate concentration is present, were seeded into 96-well plates to measure the viral titer. The sterilization test was conducted using the serial log dilution method by mixing 5 × PAW with SARS-CoV-2-infected Vero-E6 culture solution at room temperature for 10 min. Viral titer was estimated by verifying the number of wells showing the viral cytopathic effect (CPE) for each dilution in PAW-treated wells and untreated control wells. The SARS-CoV-2 titer was reduced by 4.56 log TCID_50_/mL, indicating more than 99.99% sterilization effect (Fig. 6A). Viral CPE, the observed substantial decrease in the number of SARS-CoV-2 infected cells, and cell debris due to cell death, in SARS-CoV-2 only infected cells were prominent in the light microscopy field. On the other hand, no considerable cytotoxicity was observed in PAW-only treated cells and cells treated with a mixture of SARS-CoV-2 and PAW, as compared to non-treated cells as a control. Thus, it was confirmed that PAW inhibits cell death caused by SARS-CoV-2 by a sterilizing effect on SARS-CoV-2 and does not induce cytotoxicity in normal Vero-E6 cells (Fig. 6B).

## Discussion

Plasma medicine has been an active area of research in the past few decades, with substantial evidence for its considerable sterilizing effect against various pathogens^13^. Indirect plasma-treated medium has recently shown promising effects as a disinfectant, similar to the effects of direct treatment with gas-type plasma^30^. Accordingly, studies of PAW have revealed new physicochemical properties, and evaluated as a replacement for existing sanitizing solutions in various fields, such as food and agriculture, owing to the improved flexibility and storability^18,31,32^. To further explore the application of this liquid-type plasma, we explored the use of our microwave PAW as a novel sanitizing solution as an alternative to traditional skin antiseptics.

Plasma is typically classified into thermal and non-thermal types according to the temperature, and non-thermal plasma is generally used for biomedical applications^13,33^. An advantage of indirect plasma over direct treatment is that thermal plasma can be used for the pretreatment of aqueous media, enabling more efficient chemical reactions^32^. In this study, microwave plasma, classified as thermal atmospheric plasma, was used as a plasma source. Previous studies have demonstrated that a microwave plasma torch system benefits from the generation of high-density electrons and active species^34,35^. In PAW generated by our microwave plasma system, RNS effectors represented by NO_3_^−^ generated by dissolving NO and NO_2_ as primary reactive species were maintained at high concentrations for more than 6 months, even in PAW stored at room temperature. The concentration of NO_3_^−^ in PAW was maintained higher (about 400 ppm) than that required for the complete sterilization of *S. aureus* and *E. coli*. Although the sterilization effect on each bacterial strain was evaluated 5 min after plasma treatment, disinfection proceeded rapidly, within 30 s to 1 min, for most of the colonies (Supplementary Fig. S5).

Our results demonstrate that the treatment of both gram-positive and gram-negative bacteria with PAW resulted in significant log reductions. Previous studies have demonstrated that plasma is effective against a broad spectrum of pathogens, including bacterial spores, toxins, and prions that are resistant to conventional chemical treatments^13,36^. Recent research on PAWs prepared using cold atmospheric plasma has demonstrated that PAW removes biofilms, which contribute substantially to bacterial resistance to treatment with antibiotics^17^. In this sterilization process, the activity of reactive chemical species in PAW is considered. ROS and RNS derivatives damage the cell envelope as well as intracellular components, nucleic acids, and proteins, eventually killing bacteria^19,37^. The generation of these redox molecules is influenced by each element in the plasma generation process and the working gas, and even a small change in these factors causes a large change in RNS and ROS generation via chemical reactions with the liquid and the primary reactive gas generated by plasma^12,20^. In the plasma system using air, RNS is mainly generated in PAW by active nitrogen. The reaction of N from the primary reactive gas originating from plasma-activated air and O from water forms OH^−^, NO^−^, NO_2_^−^, NO_3_^−^, and ONOOH/ONOO^−^, which play major roles in bacterial inactivation^37–39^. The method for discharging the reactive gas generated by the microwave plasma system in bubble form is suitable for effective dissociation of the primary reactive NO and NO2 into water^17,38^. The gaseous contents released by bubble breakdown cause intricate plasma-liquid interactions in multiphase environments; further studies are needed to elucidate these processes^12^.

ROS and RNS are generated together from air gas, and H_2_O_2_ with other short-lived ROS are known to contributes to the main sterilization process^19,37^. There was no measurable H_2_O_2_ in the microwave PAW used in this study. This can be explained by the fact that the post-discharge concentration of H_2_O_2_ in previous studies gradually decreases as it interacts with RNSs in PAW to form NO_3_^−^ through the reaction with NO_2_^−^ (NO_2_^−^ + H_2_O_2_ + H^+^ → NO_3_^−^ + H_2_O + H^+^)^18,39,40^. Furthermore, the sterilizing ability of PAW was maintained even after treatment with NAC, a known ROS inhibitor, indicating that ROS, including H_2_O_2_, were not the main effectors. These results are consistent with those of Naitali et al., who found that the addition of H_2_O_2_ to PAW has little effect on bactericidal activity^25^.

Conductivity increases as ROS and RNS generated by plasma treatment dissolve in water in the form of ions^18^. In addition, the reaction between chemical species formed by plasma with water also causes the acidification of the solution, and the controlled decrease in pH during PAW generation has long been presented as a major determinant of the anti-microbial effect of PAW^41^. Recent studies have consistently reported that the antibacterial effect of an acidic environment alone is limited, whereas the reactions of plasma-derived reactive species, including RNS, promoted by the acidic condition has a synergistic effect, resulting in remarkable bactericidal activity^9,15,37^. When the conductivity of the PAW was adjusted to 1000 µs/cm or greater and the pH was adjusted to 0–4, we investigated which RNSs generated in the PAW was the main effector of microbial inactivation. NO_2_^−^ and NO_3_^−^ ions in PAW have been classified as long-lived RNS, and their anti-microbial activity with the acidification of PAW has been studied extensively, since detection and quantification are relatively uncomplicated^12,18^. Using ion chromatography to analyze our microwave PAW, only NO_3_^−^ was detected, and the concentration of NO_3_^−^ was maintained for longer than 7 months. Shen et al*.* have shown that NO_3_^−^ is a long-acting biological effector of PAW for killing bacteria during storage for 30 days^20^.

ONOO^−^ is an isomer of NO_3_^−^ and exists in the form of peroxynitrous acid (ONOOH) in water, which reacts with H_2_O to produce a stable form of NO_3_^−^^42^. In this study, PAW with ONOO^−^ inhibitor resulted in a significantly weaker antibacterial effect compared with that of PAW only. ONOO^−^, an active intermediate produced during the reaction of NO_2_^−^ and H_2_O_2_ (NO_2_^−^ + H_2_O_2_ → ONOO^−^ + H_2_O) under acidic conditions, mediates a significant microbial cytotoxic effect via a strong oxidizing potential^19,43^. During the formation of ONOO^−^—in addition to H_2_O_2_—RNS reacts with other ROS such as superoxide anion radicals (·O_2_^−^) and hydroxyl radicals (·OH) (·NO + ·O_2_^−^ → ONOO^−^, ·NO_2_ + ·OH → ONOO^−^ + H^+^)^9,44,45^. Although ONOO^−^ is generated by plasma treatment, detection and measurement are challenging because it is a short-lived RNS with a half-life of less than 1 s^12^. Even in a short time, ONOO^−^ sufficiently penetrates the cell membrane and exhibits a bactericidal effect^40^. However, due to its short half-life, it is difficult to consider ONOO^−^ as the sole critical effector in the entire process of bacterial death^30^, it is necessary to further elucidate how ONOO^−^ undergoes a transformation in the cell and contributes to subsequent reactions to complete bactericidal activity. Taken together, even though ROS, RNS, and acidified environments are involved in bacterial disinfection, NO_3_^−^ isomer is the identified effector of our microwave PAW. Continuous cascades of reactions involving long-lived RNS, including the formation and isomerization of NO_3_^−^, may explain the long-term antibacterial effect of PAW^37,39^.

We proved that our microwave PAW is not cytotoxic to normal skin cells. Similar to selective cytotoxicity against bacteria, numerous studies have shown that direct or indirect treatment with plasma induces apoptosis in various types of cancer cells but has protective effects in normal cells and even promotes wound healing^46–48^. Pasqual-Melo et al*.* demonstrated the contrasting effects of plasma, which has a toxic effect on human malignant keratinocytes in cutaneous squamous cell carcinoma but promotes the proliferation of non-malignant HaCaT keratinocytes^49^. Similar to the redox-related bactericidal effect, cytotoxicity toward cancer cells is also caused by increased oxidative stress, which induces a well-known cascade of signaling pathways related to cell death^47^. Therefore, it is presumed that a difference in resistance to plasma-induced oxidative stress due to active reactive species between pathogens, cancer cells and normal skin cells explains the selective cytotoxicity. The ideal method is to establish physicochemical properties of microwave PAW that trigger a cytotoxic effect by a burst of effectors in pathogens but not in normal cells. For this, the identification of the difference in the activity of the redox molecules generated from NO_3_^−^ in different types of cells should be preceded. Thus, by determining the beneficial levels of redox molecules, controlling these levels in the PAW manufacturing process, the production of antiseptics with improved efficacy would be possible.

The mechanism by which PAW redox molecules exhibit antiviral effects while destroying the intrinsic structure of the virus has been consistently demonstrated^13,19,50^. As a novel strategy for suppressing viral dissemination after the COVID-19 epidemic since 2020, the sterilization effect of cold atmospheric plasma jet and PAW prepared with non-thermal plasma has been reported on SARS-CoV-2^51,52^. The study on the effect of PAW for SARS-CoV-2 inactivation suggested short-lived RNS as the main effector as in our study, but there is a limitation that it was conducted indirectly in a pseudovirus system using the S protein of SARS-CoV-2^52^. The experiment of this study confirmed the noticeable sterilization effect by treating microwave PAW in SARS-CoV-2 infected cell solution directly cultured in a BSL3 laboratory. A recent study presented that SARS-CoV-2 has a significantly larger RNA genome than other RNA viruses and has various potential targets for inhibitors in its proteome^53^. Thus, it can be inferred that there are targets vulnerable to redox molecules such as the NO_3_^−^ isomer in the replication process of SARS-CoV-2 in Vero-E6 cells. Although the effect was verified in the concentrated solution of PAW, unlike bacteria, it is expected that a stable, practical concentration of PAW applicable to all types of pathogens can be established by further investigation.

In conclusion, our study demonstrated the excellent antimicrobial effect of microwave PAW against bacteria and SARS-CoV-2, and at the same time, confirmed the protective effect on skin cells. It is necessary to further elucidate selective cytotoxicity and establish conditions that can stably represent the multifaceted effects of PAW. In that case, it is considered that microwave PAW will contribute as a novel disinfectant with high safety in the COVID-19 crisis as a pandemic suppression strategy in real life.

## Materials and methods

### Cell culture

Human dermal fibroblasts (HDFs) were isolated from the foreskin of patients after obtaining informed consent for the use of residual tissue for academic research. The study was approved by the Institutional Review Board of Ajou University Hospital (AJIRB-GEN-GEN-12-107) and performed following human research ethics guidelines. Normal human HaCaT keratinocytes and HDFs were cultured in Dulbecco’s modified Eagle’s medium (DMEM; Welgene, Daegu, Korea) supplemented with 10% fetal bovine serum (FBS; Gibco, Waltham, MA, USA), 1% antibiotics (Gibco, 15240-062), and 1% sodium pyruvate (Gibco, 11360-070). The cells were incubated at 37 °C with 5% CO_2_ under humidified conditions. Cells were dissociated from the cell culture surface using 0.05% Trypsin/0.02% EDTA (Invitrogen, Waltham, MA, USA) and were used from passages 2 to 5.

### Microwave plasma system and PAW generation

Figure 1A shows the 2.45 GHz microwave plasma system for generating nitric oxides (mainly, NO and NO_2_) and the system for preparing activated-water containing nitric oxides. The plasma system consisted of a microwave generator (magnetron), WR-340 waveguide components, and a microwave plasma torch. To generate the microwave plasma, 20 L/min air as a swirl gas enters the quartz tube through the quartz tube holder, forming a vortex stream in the tube. Swirl gas was adjusted using a mass flow controller (MFC). The microwave power was measured by a power monitoring system equipped with the directional coupler in Fig. 1A. The magnetron in Fig. 1A has the maximum power of 2 kW and generates microwave energy for plasma discharge. The microwave power in this experiment was operated at 1.2 kW, which was generated by air plasma torch flames (Fig. 1B). The system for preparing activated water containing nitric oxides was composed of a cooler, basins for PAW, and a neutralization vessel for residual nitric oxide gases. The hot nitric oxide gases generated in the plasma flame enters a cooler to lower the gas temperatures close to room temperature. As shown in Fig. 1A, the cooled gases are divided by two pipelines and are introduced into two PAW basins via porous alumina bubblers to increase the dissolution rate of nitric oxide gases. Water used for PAW is deionized water with electrical conductivity of about 3 µs/cm and pH of approximately 6.6, and two basins generated the 200 L of PAW. The residual gases after the dissolution in water are neutralized in a water reactor with urea (NH_2_CONH_2_). The urea water tank was installed at the rear end to remove the NO and NO_2_ gas remaining after the oxidation–reduction reaction for decrease air pollution, because NO and NO_2_ gas caused micro-dust.

### Ion chromatography analysis of PAW

NO and NO_2_ concentration in PAW was determined by using an ion chromatography system (Dionex ICS-90) in combination with an auto-sampler. The eluent was a mixture of sodium carbonate and sodium hydrogen carbonate, and the separation column was made of packed resin. After exiting the column, a suppressor was used to minimize the background conductivity of the eluent and thus enhance the detection of NO and NO_2_ ions”.

### Measurement of the conductivity of PAW

Conductivity was measured using a TPS SmartCHEMC analytical benchtop conductivity meter fitted with a low‐immersion electrochemical sensor (*K* = 1.0) and a temperature probe for temperature corrections. The pH of the PAW was measured using a double-junction, gel‐filled pH probe with a built-in temperature sensor from Hanna Instruments (HI12300; Woonsocket, RI, USA) connected to a Hanna Instruments multiparameter photometer and pH meter (HI83399).

### Measurement of RNS of PAW

To measure the nitrite concentration, the Griess assay was employed based on the reaction of *N*‐(1‐naphthyl)‐ethylene diamine hydrochloride with sulfanilic acid, resulting in the formation of a magenta‐colored azo dye with an absorption wavelength of 525 nm. A nitrate combination ion‐selective electrode from Thermo Fisher Scientific (9707BNWP; Waltham, MA, USA) was used to selectively quantify the nitrate ions present in the PAW. The probe was calibrated using reference standards and was capable of NO_3_^−^ measurements between 0.4 and 62,000 ppm. To remove the influence of interfering ions on the measurement, a nitrate interference suppressor solution was added to the sample at a ratio of 1:50.

The nitrate concentration in the PAW according to the microwave treatment time and the monthly change in the nitrate concentration of PAW over the course of 12 months were measured.

### Measurement of H_2_O_2_ in PAW

The H_2_O_2_ levels were determined using the hydrogen peroxide assay kit (BioVision, Milpitas, CA), according to the manufacturer’s protocol. Briefly, control solution, PAW or HeO_2_-treated solution was mixed with a 50 µL reaction mixture, and then they were incubated at room temperature for 10 min. Concentrations of H_2_O_2_ were determined with a microplate reader at 570 nm.

### Bacterial strains, growth conditions, and PAW treatment

*Staphylococcus aureus* (ATCC 6538), *Escherichia coli* (ATCC 11229), *Salmonella* Typhimurium (ATCC 13311), *Bacillus cereus* (ATCC 21772), and *Pseudomonas aeruginosa* (ATCC 15522) were used in the disinfection tests. Bacteria were grown at 37 °C in each bacterial medium until reaching the logarithmic phase, approximately 10^8^ CFU/mL. Then, 1 mL of the bacterial suspension was centrifuged at 5000 rpm for 10 min and resuspended in 1 mL of phosphate-buffered saline (PBS). As a control group, 0.1 mL of a bacterial solution was added to 0.9 mL of PBS and reacted at room temperature for 5 min. In the test groups, 0.1 mL of the bacterial solution was added to (PAW 0.9 mL) and reacted at room temperature for 5 min. After centrifugation at 9000 rpm for 1 min, the supernatant was removed, and 1 mL of the TSB medium or PBS was added to suspend the precipitate. Then, 0.1 mL of each suspension was spread on tryptic soy agar (TSA) plates, followed by incubation at 37 °C for 24 h. The final microbial counts determined by manual counting, were acquired from three replicates, and expressed as log (CFU number). The CFU numbers of the control groups were rounded to 10^3^. MBC is defined as the lowest concentration of NO_3_^−^ that kills 100% of the bacteria. The MBCs for *S. aureus* and *E. coli* were determined based on the absence of colonies on TSA plates^54^. For all five bacterial strains, the experiment was conducted with undiluted PAW and a 1:2 diluted solution.

### Cytotoxicity against normal skin cells

HDFs and HaCaT cells were seeded in 96-well cell culture plates at a density of 5 × 10^3^ cells/well. After 24 h, the cells were treated with PAW. Cell viability was estimated as a ratio relative to untreated cells. After 24 h, PAW was removed and medium was added, and viability was calculated using Cell Counting Kit-8 (CCK8) (NX653; Dojindo, Kumamoto, Japan) based on absorbance at 450 nm. MTT (3-(4,5-dimethylthiazol-2-yl)-2,5-diphenyl-2H-tetrazolium bromide) was purchased from Sigma-Aldrich (St. Louis, MO, USA; 98% purity). To analyze the oil solubility and spectroscopic properties of the MTT formazan product, MTT was dissolved in distilled water and ascorbic acid was added to a final concentration of 4 mg/mL. The dark violet-blue flocculent precipitate of MTT formazan was washed and resuspended in distilled water. The suspension was mixed with sunflower oil and agitated, and the oil phase was diluted with more oil. Spectrophotometric studies were performed using a UV–Vis spectrophotometer. To further evaluate cell viability, the XTT assay kit (Abcam, Cambridge, UK) was used, according to the manufacturer’s instructions. In viable cells, the 2,3-bis [2-methoxy-4-nitro-5-sulphophenyl]-2H-tetrazolium-5-carboxylanilide inner salt (XTT) is reduced by mitochondrial dehydrogenases, and this reaction leads to an orange formazan product. The supernatant was used to measure absorbance at 450 nm.

### Annexin V/PI assay

Quantitative apoptotic cell death of HaCaT cells and fibroblasts was detected using the Annexin V-propidium iodide (PI) Apoptosis Detection Kit I according to the manufacturer’s protocol (BD Biosciences, Bedford, MA, USA). Briefly, the cells were treated with PAW and incubated for 24 h. The cells were harvested, washed with cold PBS, and stained with Annexin V-fluorescein isothiocyanate and PI at room temperature for 15 min in the dark. Early and late apoptosis were quantified according to the manufacturer’s instructions. Apoptosis was detected using a FACSAria System (BD Biosciences) with excitation and emission wavelengths of 488 and 530 nm, respectively. The experiment on H_2_O_2_-treated cells as positive control was also performed in the same manner.

### Cell viability staining

The Live-Dead Cell Imaging Kit (Sigma) was used to evaluate viability in HaCaT and HDF cells. HDFs and HaCaT cells were cultured in DMEM (Welgene), 10% FBS, and 1% penicillin and streptomycin. The viability of HaCaT and HDF cells treated with PAW for 12 h was investigated by live/dead staining. Subsequently, cells were stained with calcein AM (2 μM) and ethidium homodimer-1 (4 μM) to label viable cells green and dead cells red. The cells were then incubated for 15–30 min at 37 °C. Next, cells were washed with PBS to remove the reagent for 3–5 min and the culture chambers were observed under a fluorescence microscope (EVOS2 microscope (Thermo Fisher Scientific, Waltham, MA, USA). The cells were then imaged for live cells (green fluorescence) and dead cells (red fluorescence), and the images were analyzed using ImageJ software (NIH, Bethesda, MD, USA) to quantify the cell area.

### Viral cytopathic effect (CPE) inhibition assay of PAW to SARS-CoV-2

The SARS-CoV-2 (NCCP 43326, Wuhan) sample was received from the National Culture Collection for Pathogens (NCCP) in the Korea Disease Control and Prevention Agency (KDCA). SARS-CoV-2 live culture experiments were conducted at Laboratory for Infectious Disease Prevention, Korea Zoonosis Research Institute, Chonbuk National University, South Korea in a Biosafety level 3 (BSL-3) facility. To identify the SARS-CoV-2 sterilization effect, 5 × concentrated PAW was used, which has 5000 ppm nitrate by increasing the microwave plasma treatment time five folds during PAW generation. To quantify SARS-CoV-2 infectivity, a 50% cell culture infectious dose (TCID_50_) endpoint dilution assay method was utilized^55^. Vero-E6 cells were seeded in 96-well cell culture plates at a density of 2 × 10^4^ cells/well and incubated for 16 h. After mixing 9 mL of 5 × PAW and 1 mL of SARS-CoV-2 (1 × 10^7^ TCID_50_), it was incubated at room temperature for 10 min. 1 mL of the SARS-CoV-2 mixture in a total volume of 10 mL was transferred to a separate tube, 9 mL of a cell culture solution containing 10% FBS was added, and tenfold serial dilution was performed on each recovered sample.100 μL of the diluted viral solution was added to each Vero-E6 cell well for infection. Along with the PAW-treated infected cells, untreated infected cells were maintained as controls. At 72 h postinfection, the viral CPE was determined depending on whether cell death was observed by light microscopy. For each dilution concentration, the percentage of wells exhibiting CPE was recorded, and the viral titer was calculated by TCID_50_ using Reed and Muench calculation^56^.

### Statistical analysis

All experiments were performed three times and data were analyzed. The parameter values are expressed as means and standard deviations. Group means were compared using the non-opposite *t*-test, and values of P < 0.05 (*), P < 0.01 (**), and P < 0.001 (***) were considered significant.

## Supplementary Information

Supplementary Figures.
