# Supplementary material for: Antimicrobial effects of microwave plasma-activated water with skin protective effect for novel disinfectants in pandemic era
Source: Sci Rep. 2022 Apr 8;12:5968. doi: 10.1038/s41598-022-10009-1 (PMC8992786; doi:10.1038/s41598-022-10009-1)
Supplement: Supplementary file 1 — Supplementary Figures. [file 41598_2022_10009_MOESM1_ESM.docx]

Supplementary figures

**Antimicrobial effects of microwave plasma-activated water with skin protective effect for novel disinfectants in pandemic era**

*Hye Ran Lee^a^, Yun Sang Lee^a^, Young Suk You^b^, Jin Young Huh^c^, Kangil Kim^d^, Yong Cheol Hong^e^, and Chul-Ho Kim^a,^**

**
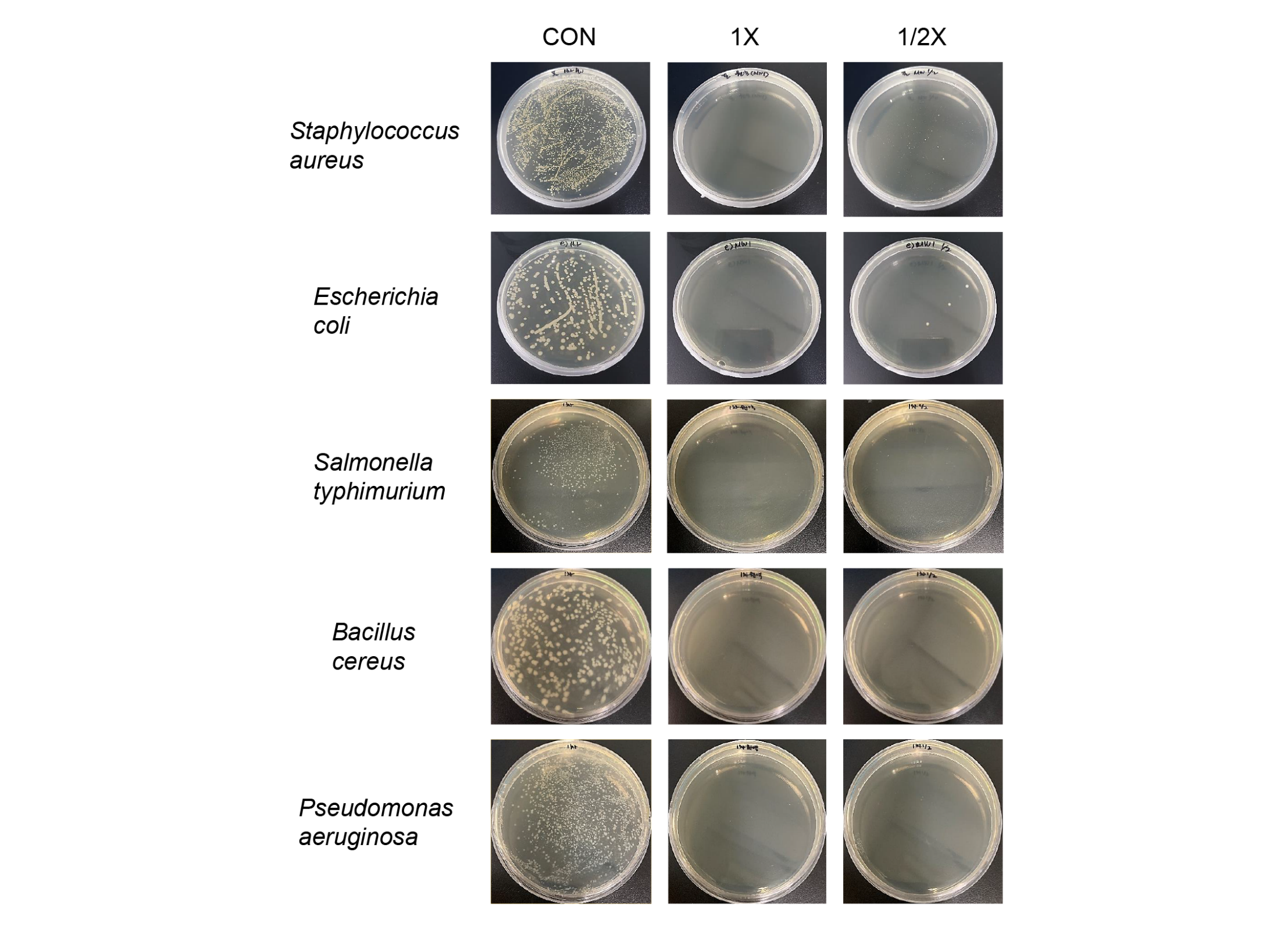
**

**Figure S1: Incubation of five bacterial strains on Tryptic Soy Agar (TSA) plates after PAW treatment.** 1× and ½× columns indicate groups treated with undiluted PAW and ½ diluted PAW solutions, respectively, and survival was compared with that in the control group treated with PBS (leftmost column).


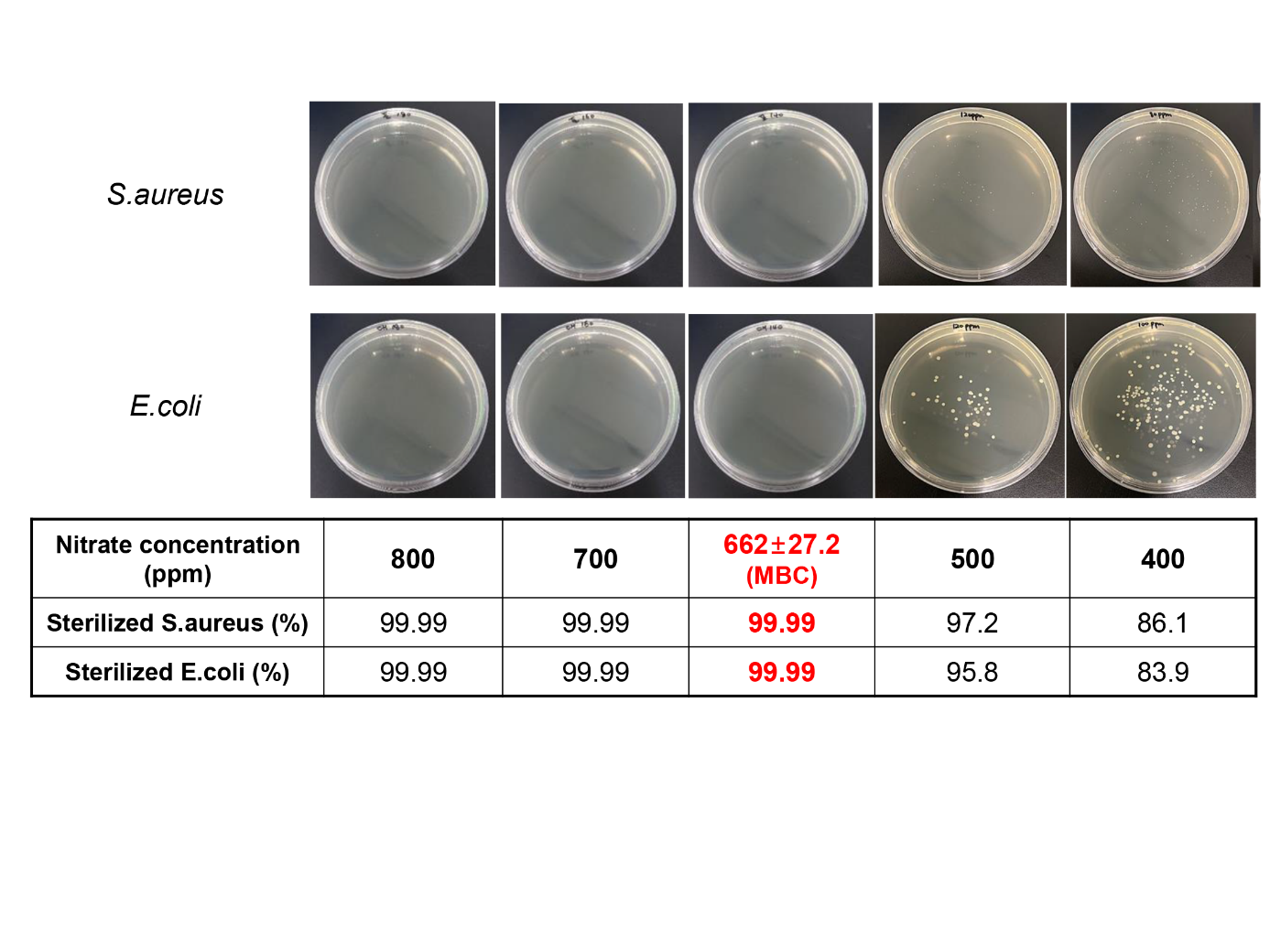


**Figure S2:** Minimum bactericidal concentration (MBC) of nitrate (NO_3_^−^) in plasma-activated water (PAW) against *S. aureus* and *E. coli*; 99.99% sterilization was achieved at 662 ± 27.2 ppm.

**
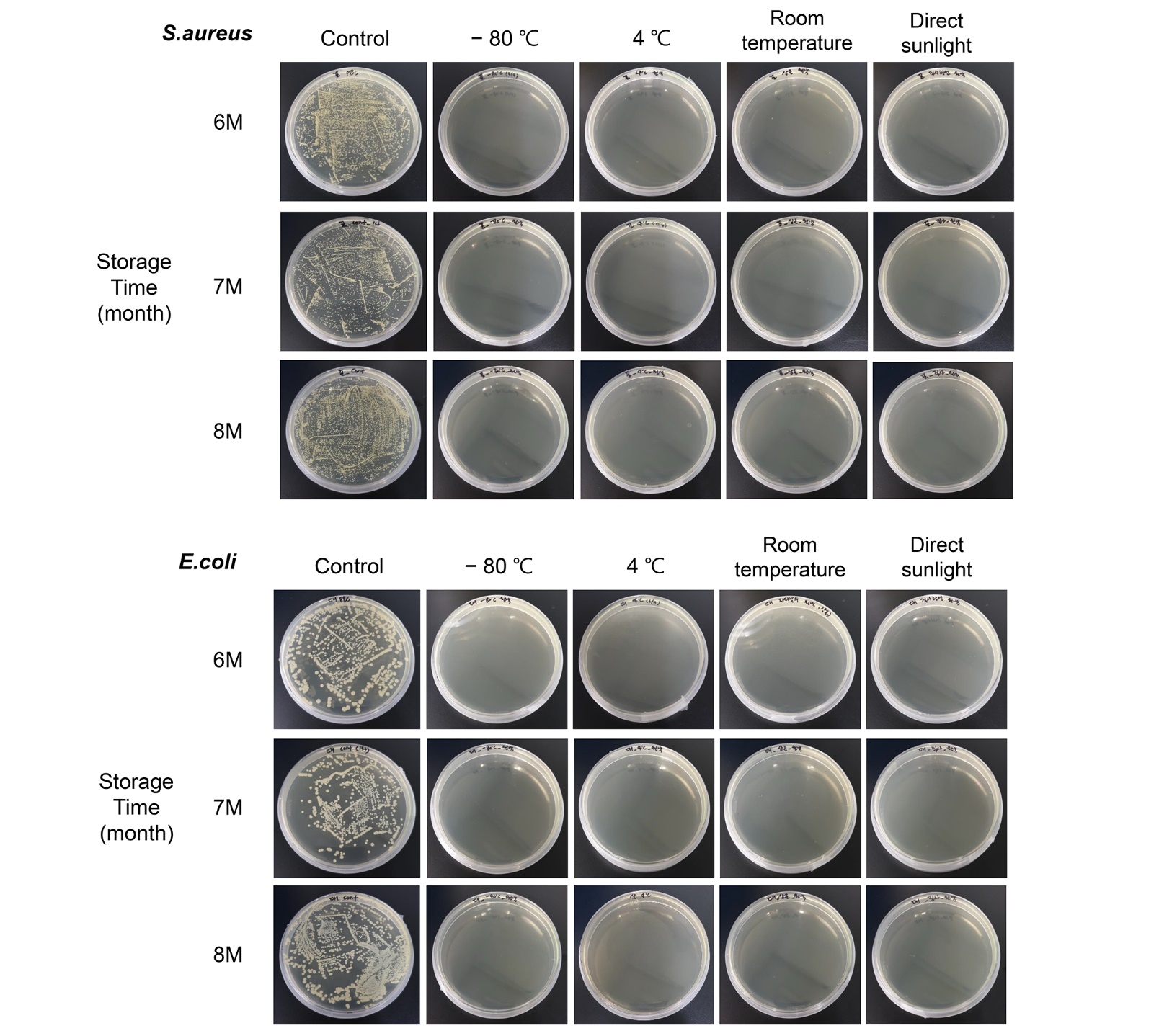
**

**Figure S3: Sterilization effect of PAW maintained under different temperature conditions.** The bactericidal effects of undiluted PAW against *S. aureus* and *E. coli* were sustained for up to 8 months at −80°C, 4°C, room temperature, and under direct sunlight.

**
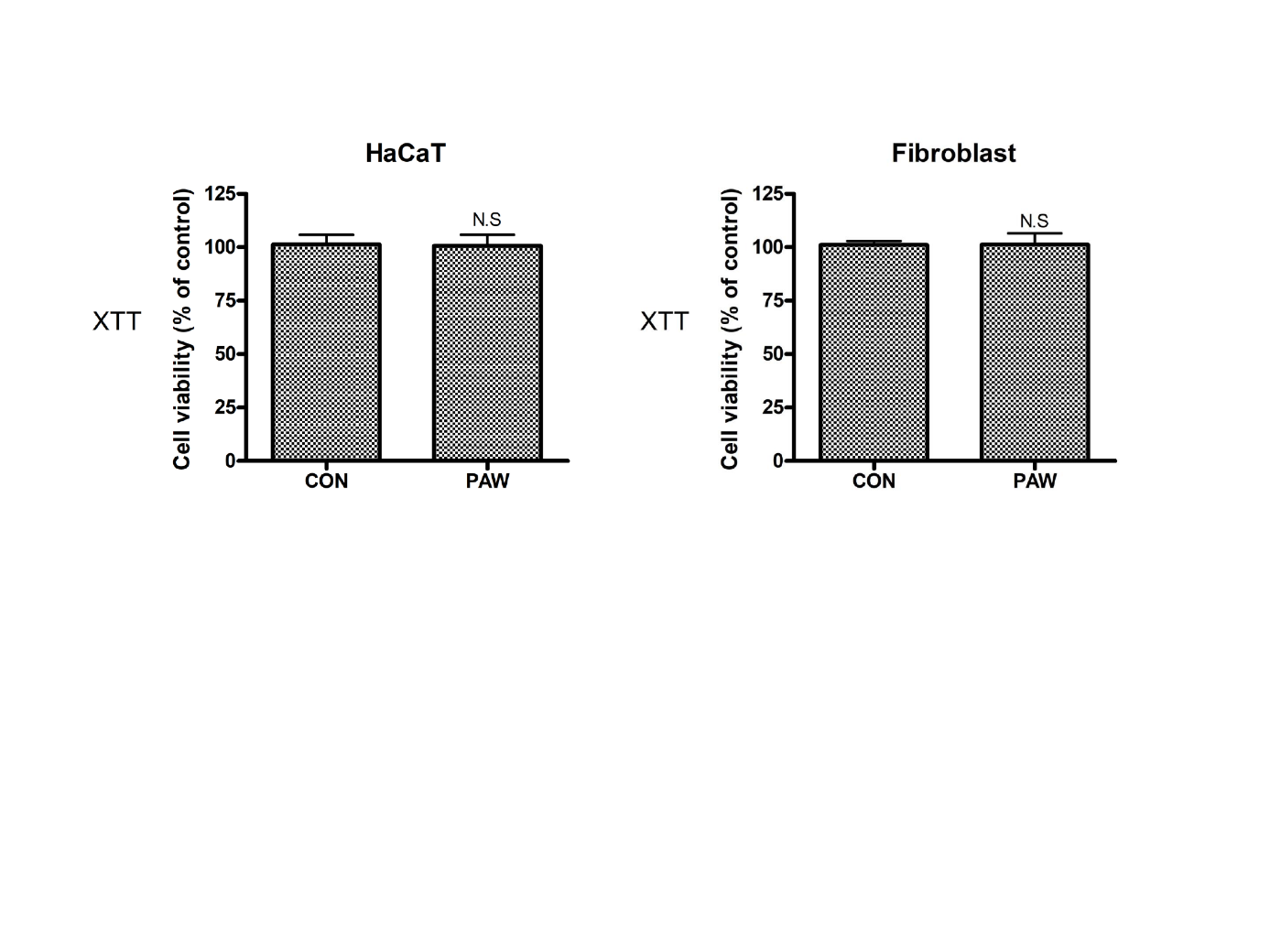
Figure S4: Summary of XTT assay results.** There was no significant cytotoxicity toward normal skin cells in the PAW-treated group compared with the control group.

**
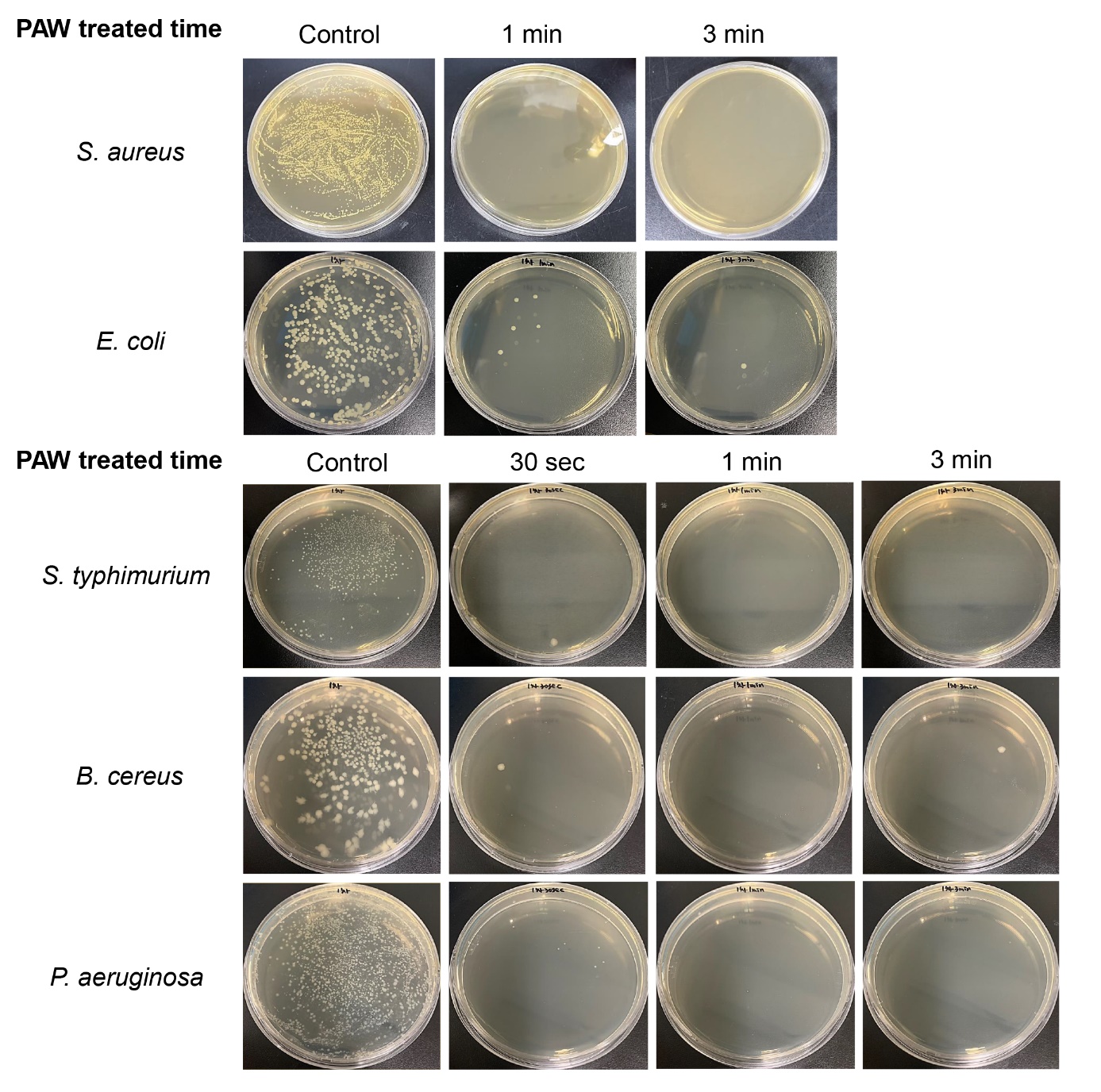
**

**Figure S5: Sterilization efficacy within 3 min of PAW treatment.** In *S. aureus*, all bacteria were killed after incubation for 1 min and 3 min. In *E. coli*, a small number of bacteria remained, even after incubation for up to 3 min. Most *S.* Typhimurium, *B. cereus*, and *P. aeruginosa* were killed within 30 s.
